# Factors associated with retention of health workers in remote public health centers in Northern Uganda: a cross-sectional study

**DOI:** 10.1186/s12960-023-00870-0

**Published:** 2023-10-17

**Authors:** Andrew Twineamatsiko, Nathan Mugenyi, Yvonne Nabachwa Kuteesa, Ejalu David Livingstone

**Affiliations:** 1 Faculty of Health Sciences, Uganda Martyrs University, Kampala, Uganda; 2 Faculty of Medicine, Mbarara University of Science and Technology, Mbarara, Uganda; 3 Abacus Parenteral Drugs Limited, Mukono, Uganda; 4 Brunel University, London, UK; 5 Seed Global Health, Kampala, Uganda; 6 Makerere University College of Health Sciences, Kampala, Uganda

**Keywords:** Health workers, Health worker retention, Hard-to-reach areas

## Abstract

**Background:**

Health worker retention in remote and hard-to-reach areas remains a threat in most low- and middle-income countries, and this negatively impacts health service delivery. The health workforce inequity is catastrophic for countries like Uganda that still has a low health worker to patient ratio, and remote areas like Lira District that is still recovering from a long-term civil war**.** This study explores factors associated with retention of health workers in remote public health centers in Lira district in Northern Uganda.

**Methods:**

A descriptive cross-sectional study with quantitative methods of data collection was used among health workers namely; doctors, clinical officers, nurses, midwives, pharmacists and, laboratory technicians. The study utilized a structured questionnaire with closed ended questions to obtain quantitative information.

**Results:**

Most of the respondents were females (62.90%), married (84.62%), with certificate level (55.74%), and nurses as qualification (36.60%) as well as attached to Health Center 3 level (61.28%). Significant individual factors associated with retention included having a certificate as highest level of education, staying with family, and working at facility for 6 or more years. The health system factors were good physical state of facility, equipment availability, availability of sundries, feeling comfortable with rotations, receiving adequate support from staff, feeling valued and respected by colleagues at workplace and access to incentives while career factors were job satisfaction, job motivation, promotion, and further training on scholarship.

**Conclusion:**

The study established that indeed several individual and social demographics, health system and career-related factors are significantly associated with retention of Health workers in the rural public health facilities and these are critical policy recommendations for establishing retention guidelines in a national human resources for health manual.

**Supplementary Information:**

The online version contains supplementary material available at 10.1186/s12960-023-00870-0.

## Background

Retention of health workers refers to efforts, policies and overall strategies to maintain staff in practice and prevent attrition [1].

Globally, World Health Organization (WHO) estimated that the world will be short of 18 million health workers to deliver universal health coverage by 2030 [2]. According to WHO (2006:8), countries with the lowest burden of disease needs have the highest number and density of health workers, while those with the highest disease burdens tend to have much lower numbers of health workers. These shortages are typically the most severe in rural areas, where almost half of the world’s population lives.

In sub-Saharan Africa, health retention workforce-related challenges remain one of the major threats to the attainment of the Sustainable Development Goal (SDG) three; including Universal Health Coverage (UHC) with the COVID-19 pandemic’s health, health systems and socio-economic impacts threaten to worsen the health outcomes of the more vulnerable rural populations [3].

Globally, half of the world’s population lives in rural and remote areas, the biggest challenge is that most health workers live and work in urban areas and cities. This imbalance that cuts across all countries and most severe in low-income countries poses a major challenge in provision of health services [4].

WHO set up several guidelines to support health systems and established policies to improve retention of the health workforce [5].

Education guidelines include targeted admission policies locating health professional institutions outside cities, expose students to rural community experiences and rotations, revise curricula to cover competency in working in rural areas as well as continuous professional development programs that address the needs of rural areas.

Regulatory guidelines include introducing different types of health workers to increase number, ensuring compulsory service requirements in rural and remote areas accompanied by appropriate support and incentives, providing scholarships with return of service in remote areas.

Financial incentives such as housing allowances, hard-to-reach allowance, free transportation as well as paid vacations.

Personal and professional support include improving living conditions in rural areas, mentorships, career development programs.

Uganda has implemented a number of policies based on the set guidelines in a bid to improve rural attraction and retention [6].

Furthermore, the Ministry of Health in the Human Resources for Health strategic plan 2020–2030 cited a number of guidelines on retention, addressing specific barriers, incentivizing hard to reach, scholarship opportunities, career development and recognition for staff working in rural and remote areas [7].

The study was conducted in Lira District, Northern Uganda, this location is remote and was hit by a long-term civil war [8]. It is still struggling with Health workforce gap currently at 67% absorption rate and had only one Doctor available in the remote health center in 2020. The rural areas house health facilities at a level of Health Center 2, 3 and 4 [9].

The district has since implemented a number of strategies to improve retention including hard-to-reach allowances, infrastructure development, awarding study leaves to long serving health workers, deploying health institution students for internship and placements in rural areas and routine support supervision [10].

There is need to study the effects of individual factors limiting retention including social factors, impact of health worker migration across districts, personal and professional factors as well as burden of upcoming disasters and epidemics such as COVID-19 on retention of health workers in rural areas. This paper explores factors associated with retention of Health workers in remote Public Health Centers in Lira district in Northern Uganda.

## Methods

### Aim

To determine factors associated with retention of health workers in remote rural public health centers in Lira district Northern Uganda.

### Study design

A descriptive cross-sectional study using quantitative method of data collection was used.

### Study setting

The study was conducted in rural public health facilities in Lira district, Lango sub-region, Northern Uganda. It is located at Latitude: 2° 19′ 60.00″N Longitude: 33° 05′ 60.00″E by coordinates.

### Study population

Data were collected from 235 Health workers in rural health centers who had worked in the District by Financial year 2021/2022 (see Table 1).

**Table 1:** Study variables

Independent variables	Dependent variable: health worker retention		
	Data to be collected	Method	Tools
Individual and social demographic factors	• Age	Quantitative	Structured questionnaire
	• Sex		
	• Marital status		
	• District		
	• Education level		
	• Qualification change of environment		
	• Proximity to families		
	• Opportunities		
Career-related factors	• Professional development		
	• Workplace environment		
	• Professional support network		
	• Promotion		
Health system factors	• Remuneration		
	• Workload		
	• Availability of supplies, medicines		
	• Infrastructure		

### Data collection tool and procedure

The study utilized a structured questionnaire with closed ended questions to obtain the quantitative information. The tool was contextualized from the Uganda Human resources for Health strategic plan [7]. Permission and ethical approval to do the study was granted prior to the study.

### Data management and analysis

Unique identifiers were used during data collection to ensure privacy of participants. Data were kept under password protected file. A database was set up using Epi data version 3.2 and data were double checked then exported to STATA version 15 for analysis. The primary outcome was retention of health workers. The measure of association was Prevalence Ratio (PR). At multi-variable levels, logistic regression model was used to independently determine the association between various independent variables and the dependent variable.

## Results

### Background information

The response rate was 100% with all the 235 participants responding to the questionnaire. The retention rate was 71.49% with 28.51% attrition. Most of the health workers were attached to HCIIIs (61.28%), females (62.90%), married (84.62%), with certificate level (55.74%), and nurses (36.60%) as indicated in Table 2 below.

**Table 2:** Demographic characteristics of participants

Variable	Category	Frequency	Percentage (%)
Total participants	Left service	67	28.51
	Still retained	168	71.49
Facility level	HC 2	23	9.79
	HC 3	144	61.28
	HC 4	68	28.94
Gender	Females	148	62.9
	Males	87	37.02
Marital status	Married	199	84.62
	Separated/divorced	9	3.85
	Single	27	11.54
Highest level of education	Bachelor	10	4.26
	Certificate	131	55.74
	Diploma	94	40
Qualification cadre	Nurse	86	36.6
	Clinical officer	23	9.79
	Doctor	10	4.26
	Laboratory technician	32	13.62
	Midwife	69	26.36
	Pharmacist	4	1.7
	Others	11	4.68

### Individual factors associated with retention among health workers

Table 3 below indicates that age group of 31–40 (PR = 0.74, *P* < 0.001), having a certificate as highest level of education (PR = 0.72, *P* = 0.008), staying with family (PR = 1.33, *P* < 0.001), and working at facility for 6 or more years (PR = 0.20, *P* < 0.001) were significantly associated with health worker’s retention at facility.

**Table 3:** Individual factors associated with retention among health workers in Lira District

Variables	Left	Retained		
	n (%)	n (%)	Prevalence ratios/PR	*P*-value
			(95% CI)	
Age				
22–30	3 (4.48)	29 (17.26)	Ref (1)	
31–40	33 (49.25)	68 (40.48)	0.74 (0.62; 0.89)	< 0.001
41–50	25 (37.31)	54 (32.14)	0.75 (0.63;0.91)	0.003
51 years +	6 (8.96)	17 (10.12)	0.82 (0.62;1.07)	0.136
Level of education				
Bachelor	1 (1.49)	9 (5.36)	Ref (1)	
Certificate	46 (68.66)	85 (50.60)	0.72 (0.57;0.92)	0.008
Diploma	20 (29.85)	74 (44.05)	0.87 (0.69;1.10)	0.259
Qualification cadre				
Nurse	26 (38.81)	60 (35.71)	Ref (1)	
Clinical officer	8 (11.94)	15 (8.93)	0.93 (0.67;1.30)	0.689
Doctor	1 (1.49)	9 (5.36)	1.29 (1.01;1.66)	0.046
Laboratory technician	7 (10.45)	25 (14.88)	1.12 (0.89;1.41)	0.336
Midwife	24 (35.82)	45 (26.79)	0.93 (0.75;1.17)	0.551
Pharmacist	1 (1.49)	3 (1.79)	1.08 (0.60;1.93)	0.808
Others	0 (0.00)	11 (6.55)	1.43 (1.25;1.65)	< 0.001
Distance from workplace to home				
Within 5 km	7 (10.45)	81 (48.21)	Ref (1)	
6–13 km	7 (10.45)	23 (13.69)	0.83 (0.68;1.02)	0.084
14–30	28 (41.79)	28 (16.67)	0.54 (0.41;0.71)	< 0.001
31–60	22 (32.84)	29 (17.26)	0. 62 (0.48;0.79)	< 0.001
60 plus km	3 (4.48)	7 (4.17)	0.76 (0.50;1.15)	0.192
Staying with family				
No	55 (82.09)	98 (58.33)	Ref (1)	
Yes	12 (17.91)	70 (41.67)	1.33 (0.56;0.72)	< 0.001
Period worked at health facility				
≤0.5 years	0 (0.00)	10 (5.95)	Ref (1)	
0.6–1 years	7 (10.45)	21 (12.50)	0.75 (0.61;0.93)	0.009
2–5 years	9 (13.43)	124 (73.81)	0.93 (0.89;0.98)	0.003
6 or more years	51 (76.12)	13 (7.74)	0. 20 (0.12;0.33)	< 0.001

### Association between health system factors and retention of health workers

From Table 4 below, health system factors such as good physical state of facility (PR = 3.15, *P* = 0.007), equipment availability (PR = 0.69, *P* < 0.001), availability of sundries (PR = 0.69, *P* < 0.001), feeling comfortable with rotations (PR = 1.67, *P* < 0.001), receiving adequate support from staff (PR = 2.70, *P* < 0.001), feeling valued and respected by colleagues at workplace (PR = 5.97, *P* = 0.001) and access to incentives (PR = 1.92, *P* < 0.001) were significantly associated with retention of health workers.

**Table 4:** Association between health systems factors and retention among health workers in Lira District

Variable	Left	Retained	values	
	n (%)	n (%)	PR (95% CI)	*P*-Values
Physical state of the facility				
Poor	10 (14.93)	4 (2.30)	Ref (1)	
Moderate	50 (74.63)	101 (60.12	2.34 (1.01;5.41)	0.047
Good	7 (10.45)	63 (37.50)	3.15 (1.37;7.25)	0.007
Equipment availability				
Chronic lack of equipment	4 (5.97)	30 (17.86)	Ref (1)	
All equipment available	7 (10.45)	3 (1.79)	0.69 (0.63;0.76)	< 0.001
Some equipment available	56 (83.58)	135 (80.36)	0.76 (0.64;0.90)	0.002
Working hours (more than 40 h a week)				
Never	0 (0.00)	19 (11.31)	Ref (1)	
Sometimes	14 (20.90)	120 (71.43)	0.90 (0.85;0.95)	< 0.001
Most times	53 (79.10)	29 (17.26)	0.35 (0.26;0.47)	< 0.001
Do you feel comfortable with the rotations/transfers				
No	35 (52.24)	33 (19.64)	Ref (1)	
Yes	32 (47.76)	135 (80.36)	1.67 (1.29;2.15)	< 0.001
Receiving adequate support from staff				
Does not receive adequate support	36 (53.73)	16 (9.52)	Ref (1)	
Receive adequate support	31 (46.27)	152 (90.48)	2.70 (1.78;4.08)	< 0.001
Feeling valued and respected by colleagues at workplace				
No	20 (29.85)	3 (1.79)	Ref (1)	
Yes	47 (70.15)	165 (98.21)	5.97 (2.07;17.22)	0.001
Sundries availability				
Always available	0 (0.00)	8 (4.76)	Ref (1)	
Sometimes	57 (85.07)	129 (76.79)	0.69 (0.63;0.76)	< 0.001
Very often	10 (14.93)	31 (18.45)	0.76 (0.64;0.90)	0.002
Access to incentives (housing, transport, hard-to-reach pay)				
No access	65 (97.01)	68 (40.48)	Ref (1)	
Have access	2 (2.99)	100 (59.52)	1.92 (0.43;0.60)	< 0.001

### Career-related factors and retention among health workers

Table 5 below indicates the career factors that are significantly associated with retention, these include job satisfaction (PR = 1.49, *P* < 0.001), job motivation (PR = 1.49, *P* < 0.001), promotion (PR = 1.33, PR < 0.001), further training on scholarship (PR = 1.45, *P* < 0.001). Others include measures to prevent harassment at workplace, clear and fair job appraisal, prevention of injuries and social conflicts at workplace as well as flexibility for personal work balance.

**Table 5:** Association of career-related factors and retention of health workers in Lira District

Variables	Left	Retained		
	n (%)	n (%)	Prevalence ratios PR (95% CI)	*P*-value
Job satisfaction				
Not satisfied	41 (61.19)	100 (59.52)	Ref (1)	
Moderately satisfied	26 (38.81)	17 (10.12)	0.56 (0.38;0.82)	0.003
Very satisfied	0 (0.00)	51 (30.36)	1.49(1.27;1.57)	< 0.001
Motivation to work				
Not motivated	30 (44.78)	61 (36.31)	Ref (1)	
Very motivated	0 (0.00)	39 (23.21)	1.49 (1.29;1.72)	< 0.001
Moderately motivated	37 (55.22)	68 (40.48)	0.97 (0.79;1.18)	0.738
Promotion after a period of time at work				
No	56 (83.58)	102 (60.71)	Ref (1)	
Yes	11 (16.42)	66 (39.29)	1.33 (1.15;1.54)	< 0.001
Opportunity for in service training				
No	42 (62.69)	85 (50.60)	Ref (1)	
Yes	25 (37.31)	83 (49.40)	1.15 (0.98;1.35)	0.091
Assurance of further training and specialization on a scholarship				
No	63 (94.03)	114 (67.86)	Ref (1)	
Yes	4 (5.97)	54 (32.14)	1.45 (1.27;1.65)	< 0.001
Measures to prevent harassment at work				
No	35 (52.24)	14 (8.33)	Ref (1)	
Yes	32 (47.76)	154 (91.67)	2.90 (1.85;4.54)	< 0.001
Having a clear and fair job appraisal				
Never	0 (0.00)	49 (29.17)	Ref (1)	
Most times	46 (68.66)	89 (52.98)	0.66 (0.58;0.74)	< 0.001
Sometimes	21 (31.34)	30 (17.86)	0.59 (0.47;0.74)	< 0.001
Flexibility to balance the demand of the workplace and personal				
No	51 (76.12)	19 (11.31)	Ref (1)	
Yes	16 (23.88)	149 (88.69)	3.33 (2.26;4.90)	< 0.001
Having specific measures to protect against workplace injuries				
No	30 (44.78)	14 (8.33)	Ref (1)	
Yes	37 (55.22)	154 (91.67)	2.53 (1.63;3.93)	< 0.001
Having specific measures to prevent social conflicts in the workplace				
No	46 (68.66)	28 (16.67)	Ref (1)	
Yes	21 (31.34)	140 (83.33)	2.30 (1.70;3.10)	< 0.001
Getting socially recognized and rewarded				
Most times	13 (19.40)	30 (17.86)	Ref (1)	
Never	15 (22.39)	57 (33.93)	0.97 (0.77;1.22)	0.781
Once in a while	39 (58.21)	81 (48.21)	1.13 (0.90;1.43)	0.282

## Discussion

### Retention rate of health workers

The retention rate of health workers in remote rural public health centers was 71.49%. This is low considering the health workforce demand of the District and Uganda that has doctor–patient ratio estimated at 1:25,725 and the nurse-to-patient ratio at 1:11,000 [11]. This retention rate could be attributed to COVID-19 pandemic that had a significant effect on health workforce in the country and globally [12].

Several developing countries also have low retention rates for example in Guinea, 69% of the officially deployed health workers were retained after 1 year [13], and 85% in Cameroon after a 3-year period of follow-up [14] and Thailand with 78.2% [15].

In accordance with the background of respondents, retention was significantly high among the workers at Health Center 3 level, certificate as the highest level of education and the Nurses. Studies have pointed out the fact that rural entities are filled with staff holding lower levels of education may be due to the search for greener pastures by highly qualified unless (in some cases) when the person originates from that particular area [16].

### Factors associated with retention among health workers

#### Individual factors

Among the individual factors, age group of 31–40 (PR = 0.74, *P* < 0.001) meaning prevalence of retention was 0.74 times among the 31–40 age group than the 22–30 reference group, shorter distance from facility to parental home (PR = 0.69, *P* < 0.001), staying with family at workplace (PR = 1.33, *P* < 0.001) in which the health workers that stayed with their families had a prevalence of retention being 1.33 times than those that did not stay with their families, and staying longer at the same facility (PR = 0.41, *P* < 0.001) were significantly associated with retention.

A study in Tanzania indicated that an age of below 35 years was positively associated with retention and this could be because a relatively increased age is associated with strong motivation and commitment to work [17]. Another study also indicated that an increased age is positively associated with retention [18].

A related study conducted in Nigeria noted the significant role of staying with family at workplace as a strong factor for retention which relates to our findings, this increases family bondage and limits desire for health workers to change employment posts in search of family proximity [19]. In regard to effect of staying longer at the facility, noted that length of stay has an overall influence on retention as health workers tend to establish their lives and future with in the specific community [20].

#### Health system factors

Among the health system factors, the physical state of the health facility (PR = 3.15, *P* = 0.007) was significantly associated with retention, a good state of facility motivated health workers to stay longer at the health facilities. Health workers who worked in health facilities with good infrastructure were more likely to be retained than health workers who worked in health facilities with poor infrastructure. These findings just like other studies that indicated the role of physical state of the facility towards retention of health workers in rural areas in Kakamega, Kenya [21] and Ghana [22].

Availability of equipment was also significantly associated with retention of health workers. These results are like other studies; for instance, a study found out that availability of equipment enables the health workers to perform their duties with minimal stress hence improves patient care and willingness to stay [19]. Doctors and nurses in Bangladesh highlighted the role of good infrastructure, equipment, financial incentives and social support [23].

Those who had access to financial and non-financial incentives had a prevalence of retention at 1.92 times than those that did not have. These incentives include salary, retirement/pension (PR = 1.34, *P* = 0.016), and accommodation (PR = 1.34, *P* < 0.001). They provide a better quality of living for the health worker while at the facility and even after retirement and hence assurance of such will enable health workers to stay longer at facilities [24]. Several other studies highlight the role of financial and non-financial incentives towards retention of health workers in rural areas; in Tanzania [25] and among the health workers in Zambia [26]. A study conducted in Bangladesh showed that poor housing facilities, insufficient wages were responsible for health workers choice to leave service in rural health facilities [23].

Those that had enough staff at facility were 0.68 times more likely to be retained than those that had few, support from staff and respect from colleagues are factors that provide a good working environment, with increased staff, there would not be exhaustion, staff would work relatively normal working hours. The support and respect also promote co-development at workplace. The cross-sectional survey conducted in China to investigate what could lead to the loss of rural doctors indicated that poor socialization with others and working for more than 50 h/week were risk factors [27]. Working conditions (number of staff and working hours) were also identified as key factors in studies on retention in Sierra Leone [28] and Niger [29]. The longer working hours lead to physical and mental fatigue which escalates the health workers decision to leave such a service in search for a more accommodative and flexible work environment.

#### Career-related factors

Job satisfaction, motivation, and assurance of promotion motivate health workers to keep working at facilities as they see themselves attaining higher positions as time passes by. Promotion was highlighted in a related study in Kenya as a key factor to retention of health workers [30]. A similar study done in Uganda by indicated that formal promotions were highly important [31]. Nurses who had significant overall job satisfaction in Papua New Guinea were more likely to be retained as these inter personal factors were an intrinsic trigger [32].

Being offered scholarship and an assurance for further training were significantly associated with retention. This offers the health workers an opportunity to advance in their career. Offering such an opportunity would keep health workers much longer at the facility. This has also been noticed in several studies; among health workers in Ghana hospitals [22], and a meta-analysis to identify factors associated with health workforce retention [33].

Professional development is a key health workforce indicator and trigger for retention across all cadres not only in developing countries, but also developed countries as highlighted in a study in Australia [34].

Other factors such as preventive measures on harassment at work, a clear and fair appraisal for job performance, flexibility to balance work and personal demands, protective measures against injury at work and protective measures against social conflict at work were all highly associated with retention; they create an environment where a health worker is protected from any harm and social conflicts. For instance, health workers who were supported during the COVID-19 pandemic and given protective gears across the globe were most likely to continue working with in the health workforce [35].

These were also noted in a number of other studies, health workers in rural areas in Nigeria cited the need to balance work and personal demands [19], in a study for job satisfaction and morale in the Ugandan health workforce noted the need for a better work environment [36]. A study on Retention of Health workers in Malawi indicated that performance appraisals and job description availability is a strong motivation for health workers retention [37].

## Conclusion

The study established that indeed several individual and social demographics, health system and career-related factors are significantly associated with retention of Health workers in the rural public health facilities.

The individual factors included age, staying with family at workplace and staying longer at the facility. The health system factors included physical state of the facility, availability of equipment and sundries, access to incentives, normal working hours, increased staff at facility, support, and respect from colleagues at workplace. The career-related factors included job satisfaction, motivation to work, assurance of promotion, scholarships for further training, flexible work demand and personal lives, fair appraisal, and job description as well as protection from injuries and conflicts at workplace.

These findings are critical and key for national human resources for health policy formulation and recommended for inclusion while formulating rural health workforce retention guidelines.

## Study limitations

The study was purely quantitative and hence did not capture in-depth health worker thoughts on several factors for retention. The study could also not measure all the components of health system pillars.

## Areas of further research

A qualitative study to compliment these quantitative measurements and an in-depth study on relationship between specific factors such as leadership and governance, health workforce, medical products, vaccines and technologies, health information systems and health workforce retention in rural areas.

### Supplementary Information

**Additional file 1.** Dataset.

## Data Availability

All data generated or analyzed during this study are included in this published article as Additional file 1.

## Abbreviations

WHO: World Health Organization
EAC: East Africa Community
UN: United Nation
MOH: Ministry of Health
SDG: Standard Development Goals
UHC: Universal Health Coverage
HC: Health Center
HRH: Human Resources for Health
